# Associations of walking impairment with visual impairment, depression, and cognitive function in U.S. older adults: NHANES 2013–2014

**DOI:** 10.1186/s12877-022-03189-y

**Published:** 2022-06-06

**Authors:** Wei Gao, Pengfei Dai, Yuqian Wang, Yurong Zhang

**Affiliations:** 1 Department of Ophthalmology, Xi’an People’s Hospital (Xi’an Fourth Hospital), 21 Jiefang Road, Xi’an, Shaanxi 710061 China; 2 Department of Neurology, The First Affiliated Hospital of Xi’an Jiaotong University, 277 Yanta West Road, Xi’an, Shaanxi 710061 China

**Keywords:** Cross-sectional study, Walking impairment, Depression, Visual impairment, Cognitive function, Older adults

## Abstract

**Background:**

Walking impairment, a common health problem among older adults, has been linked to poor vision and mental health. This study aimed to investigate the associations of walking impairment with visual impairment, depression, and cognitive function in older adults.

**Methods:**

A total of 1,489 adults aged 60 years and older who had participated in the National Health and Examination Survey (NHANES) 2013–2014 in the United States were included. Multivariate logistic regression models were used to examine the associations of walking impairment with visual impairment, depression, and four subdomains of cognitive function. Sample weights were used to ensure the generalizability of the results.

**Results:**

Among all the participants (median age = 68 years; 53.7% women), 17.5% reported walking impairment. Walking impairment was significantly associated with visual impairment (adjusted odds ratio [aOR] = 2.76; 95% CI: 1.47–5.20) and depression (aOR = 4.66; 95% CI: 3.11–6.99). Walking impairment was only associated with the Digit Symbol Substitution (DSST) subdomain of cognitive function in total participants (aOR = 0.97; 95% CI: 0.95–0.99) and in non-Hispanic white adults (aOR = 0.96; 95% CI: 0.94–0.98). Participants with two or three impairment indicators had a higher OR of walking impairment (aOR = 3.64, 95% CI = 2.46–5.38) than those with 0–1 (reference group) impairment indicator.

**Conclusions:**

Walking impairment was associated with visual impairment, depression, and cognitive impairment in American older adults and also positively associated with the number of impairment indicators. The association between walking impairment and cognitive impairment varied according to race. Evaluations of vision, cognition, and depression should be conducted among older adults with walking impairment, and the needs of older adults should be provided in the evaluations alongside information on the biological aspects of their particular race.

**Supplementary Information:**

The online version contains supplementary material available at 10.1186/s12877-022-03189-y.

## Background

Older adults are the fastest-growing percentage of the United States (U.S.) population [1]. It has been estimated that the number of older adults with one or more disabilities will triple by 2050 [2]. Walking impairment affects millions of older adults worldwide and is a major cause of disability. It is associated with decreased quality of life, increased dependency on others and healthcare costs, and early mortality [3, 4]. Since the physical body deteriorates with age, older adults are more likely to experience walking impairments than any other age group [5]. Walking is a fundamental motor task essential for healthy and active living. The health benefits of walking for older adults are well documented [6, 7]. Moderate levels of walking have been shown to reduce the symptoms of depression and anxiety [8], protect older individuals from dementia and cognitive decline [9], and assist in the management of chronic diseases such as diabetes and uncontrolled hypertension [10, 11]. Several studies have reported that the gait parameters (unsteady gait and gait speed) were positively associated with visual acuity, depression, and cognitive function [12–14], while some revealed inconsistent findings [15, 16].

The burden of visual impairment, depression, and cognitive impairment will increase dramatically in the coming decades due to the aging of the U.S. population. Approximately 6.5 million Americans aged 65 years or older had severe visual impairment in 2000, and this number has been projected to double by 2030 [17, 18]. Visual impairment such as low visual acuity or visual field is associated with difficulties in walking and climbing steps [19]. Individuals with visual impairment are more likely to experience walking disabilities when they are older [20]. Depression has a strong association with infrequent walking [21], which alone or in combination with other chronic diseases can lead to a shortened active life expectancy [22]. Additionally, no significant difference has been found between walking (walking endurance, gait speed) and depression [16]. Another common chronic condition is cognitive decline, which increases with age and imposes a heavy burden on older adults living in the community [23]. A previous study showed that ordinary, frequent walking had a protective effect on older individuals with dementia and cognitive decline [9]. Exercise, including walking, can improve cognitive function in older adults with mild cognitive impairment [24], and older adults with relatively slower walking speeds are at a greater risk of developing dementia [25]. However, no significant associations have been found between gait speed and vision and executive functions [15]. In addition, the effects of racial divergence on the relationship between cognition and walking impairment are inconsistent. A study showed a curvilinear U-shaped relationship in Whites and an inverse relationship in African Americans [26], and another study revealed positive associations in Asians and Whites [27].

Studies about the associations between walking impairment and visual impairment, depression, and subdomains of cognition, either considered separately or in combination, have not fully focused on older adults, especially regarding their racial/ethnic disparities. Furthermore, although older adults often have more than one impairment indicator [28, 29], few studies have assessed the association between the combined impairments and walking impairment. Therefore, our study investigated these associations in older adults in the U.S. using data from the National Health and Nutrition Examination Survey (NHANES).

## Methods

### Study participants

**Fig. 1:**
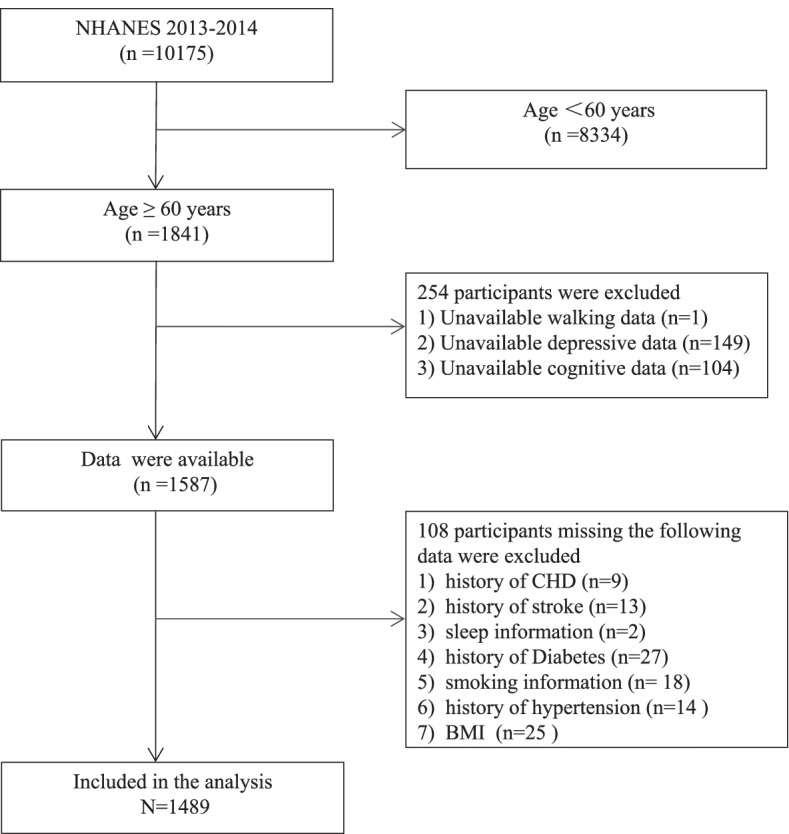
Flow chart of participant selection

The current study was a cross-sectional analysis of secondary data from the NHANES 2013–2014. The NHANES is a nationally representative cross-sectional survey of the non-institutionalized US population aged 2 years or older with data collected in 2-year cycles. During each cycle, the NHANES is conducted based on a stratified multi-stage probability sampling design and includes two components: a household interview and a health examination. Additional information on the design and procedures of the NHANES is available at the Centre for Disease Control and Prevention (CDC) website (https://wwwn.cdc.gov/nchs/nhanes/). For this study, we included individuals aged 60 years or older who had participated in the 2013–2014 NHANES. This included the most recent data on walking impairment, depressive symptoms, visual impairment, and cognitive function that were released for public use. We excluded participants who had declined to answer or had missing data, including depressive symptoms, cognitive function, visual impairment, or walking impairment. The final sample size in the analysis was 1,489 (Fig. 1). All participants provided informed consent before the study. This study was approved by the Research Ethics Review Board of the National Center for Health Statistics (NCHS) and was performed in accordance with the principles of the Declaration of Helsinki and all its amendments. The NHANES database is open to the public, and access to it does not require any ethical or administrative permission.

### Assessment of walking impairment and vision impairment

Walking impairment was assessed using the data collected with the NHANES medical condition questionnaire. Participants were classified as having a walking impairment if they reported serious difficulty in walking or climbing stairs and as having visual impairment if they reported being blind or having serious difficulty seeing even when wearing glasses. The disability questionnaire was administered at home by trained interviewers using a computer-assisted personal interview (CAPI) system.

### Depressive symptoms and cognitive function

Depression was measured using the Patient Health Questionnaire (PHQ-9), which is a commonly used screening instrument for depression, with nine items that ask about the frequency of depression symptoms in the past two weeks. The total score of the PHQ-9 ranges from 0 to 27 with a higher score indicating a greater likelihood of having a major depressive disorder. Based on the literature, we used a score > 10 as the cutoff for depression diagnosis in the current study [30, 31].

Cognitive performance was evaluated across four cognitive subdomains, primarily related to working memory, language, processing speed, and executive function: (1) the Consortium to Establish a Registry for Alzheimer’s Disease (CERAD) Total test, a measure of word learning and immediate recall module, that consists of three consecutive tests where participants are instructed to read and recall ten words in each test (scores from all three test repetitions are summed and the total score ranges from 0 to 30); (2) the Animal Fluency (AF) test, a measure of verbal fluency, which is a component of executive function, where participants are asked to name as many animals as possible in one minute; (3) the Digit Symbol Substitution Test (DSST), a measure of processing speed, sustained attention, and working memory, in which participants have two minutes to match (pair) symbols to number; and (4) the CERAD Delayed Recall (CERAD–DR) test, which provides a measure of delayed memory, where participants are asked to recall the ten words used in the CERAD Total test after the Animal Fluency and DSST tests were completed (scores range from 0 to 10). Higher scores reflect better cognitive functioning to the four subdomains tests. A score of ≤ 28 points on the DSST indicates poor or impaired cognitive performance [32].

### Covariates

The variables included age (years), sex (women vs. men), race/ethnicity (Hispanic American, non-Hispanic White, non-Hispanic Black, or other), education (less than high school, high school, or above), smoking status (current, former, or never), body mass index (BMI) (normal weight: BMI < 25; overweight: BMI 25–29.9; obese: BMI ≥ 30 kg/m^2^), drinking (having at least 12 alcohol drinks per year or not)[33], marital status (married or living with a partner, widowed/separated/ divorced, or never married), and sleep disorder. Participants’ history of diseases was recorded, including hypertension, diabetes, coronary heart disease, and stroke. Physical activity status was not included due to its high correlation with walking impairment.

BMI was calculated as the weight (kg) divided by the square of the height (m^2^). Drinking was identified as to whether the participant had at least 12 drinks of any type of alcoholic beverage in one year. A drink meant a 12 oz. beer, a 5 oz. glass of wine, or one and a half ounces of liquor. Data on sleep disorder and history of hypertension, coronary heart disease, or stroke were obtained from the questionnaire and defined as “ever been told by a doctor or other health professional”. History of diabetes was based on participants’ self-reported diagnosis of diabetes. Additionally, participants who did not report a diabetes diagnosis but had a fasting HbA1c greater than 6.4% were also considered to have diabetes.

### Statistical analysis

Descriptive analyses were conducted to summarize the participants’ characteristics. Continuous variables are described as medians with interquartile ranges (IQRs) or as means with standard deviations (SDs) if the variables had a normal distribution. Categorical variables are presented as numbers with percentages. One-way analysis of variance and chi-square tests were conducted to compare the continuous and categorical variables, respectively. The prevalence of walking impairment was estimated in all participants and by age group (60–69, 70–79, ≥ 80 years). Binary logistic regression analysis was used to investigate the associations of walking impairment with visual impairment, depression, and cognitive deficits. Depressive symptoms were analyzed both as categorical and continuous variables. All the binary logistic regression analyses were adjusted for age, sex, race/ethnicity, education, BMI, marital status, and sleep disorder (Model 1), further adjusted for medical history, which included diabetes, hypertension, and stroke (Model 2), and additionally adjusted for depression, cognitive or visual impairment (Model 3). Additionally, we performed stratified analyses according to race/ethnicity. No difference was observed between genders (Supplementary Table 1). Data were weighted to ensure that they were representative of the U.S. population using complex survey sampling analysis methods. All statistical analyses were performed using SAS software (version 9.4; SAS Institute, Cary, NC, USA). A two-tailed test was performed, and statistical significance was defined as *p* < 0.05.

## Results

This study included 1,489 participants. The median age of the total group was 68 years (interquartile range, 63–74 years), and 53.7% of the participants were women. Of the 1,489 participants, 320 (17.5%) had a walking impairment, 103 (5.3%) had visual impairment, and 155 (8.9%) had depression. Table 1 presents the characteristics of the study population stratified by their walking impairment status. Compared with those without a walking impairment, participants with a walking impairment were more likely to be widowed, separated, divorced, obese, had sleep disorders, hypertension, stroke, visual impairment, depression, and cognitive impairment, and were less likely to be educated. Older individuals were more likely to have a walking impairment: the prevalence of walking impairment was 16.1%, 17.6% and 22.9 in the 60–69 years old, 70–79 years old, and ≥ 80 years old age groups, respectively.

**Table 1:** Characteristics of study participants by walking impairment status, NHANES 2013–2014

Characteristic	Total (*n* = 1,489)	No walking impairment(*n* = 1,169)	Walking impairment (*n* = 320)	*p* value
Age, in years (median, IQR)^a^	68 (63–74)	68 (63–74)	69 (64–76)	0.363
Age, n (%)^b^				0.242
60–69 y	801 (56.3)	636 (57.3)	165 (51.8)	
70–79 y	448 (30.0)	353 (30.0)	95 (30.3)	
≥ 80 y	240 (13.7)	180 (12.8)	60 (17.9)	
Female, n (%)^b^	774 (53.7)	597 (52.4)	177 (59.9)	0.063
Race, n (%)^b^				0.248
Hispanic American	284 (7.3)	195 (6.1)	89 (12.8)	
Non-Hispanic White	760 (79.6)	611 (81.1)	149 (72.5)	
Non-Hispanic Black	306 (8.4)	245 (8.2)	61 (9.5)	
Other	139(4.8)	118 (4.7)	21 (5.2)	
H.S. Education, n (%)^b^	1151 (85.5)	934 (87.5)	217 (76.5)	0.003
Marital status, n (%)^b^				0.010
Married or living with partner	881 (65.0)	718 (67.5)	163 (53.1)	
Widowed/separated/divorced	527 (30.6)	391 (28.2)	136 (41.9)	
Never married	81 (4.4)	60 (4.3)	21 (5.0)	
Current smoking, n (%)^b^	183 (20.3)	140 (19.5)	43 (23.5)	0.361
Alcohol drinks/year, n (%)^b^				0.124
≥ 12	1021(73.4)	815(74.8)	206(61.0)	
< 12	464(26.6)	354(25.2)	110(33.0)	
Sleep disorder, n (%)^b^	181 (12.5)	108 (9.8)	73 (25.1)	< 0.001
BMI (kg/m^2^) (median, IQR)^a^	28 (25–32)	28(25–32)	32 (27–38)	< 0.001
BMI, n (%)^b^				< 0.001
Normal weight (BMI < 25 kg/m^2^)	394 (25.1)	339 (27.1)	55 (15.7)	
Overweight (BMI25-29.9 kg/m^2^)	546 (36.5)	454 (38.9)	92 (25.7)	
Obesity (BMI ≥ 30 kg/m^2^)	549 (38.4)	376 (34.1)	173 (58.7)	
Cardiovascular risk factors, n (%)^b^				
Diabetes	340 (19.6)	234 (17.5)	106 (29.3)	0.067
Hypertension	947 (60.9)	697 (57.0)	250 (79.0)	< 0.001
Stroke	99 (6.5)	66 (5.6)	33 (10.5)	0.013
Coronary heart disease	150 (10.6)	104 (9.3)	46 (16.8)	0.804
Visual impairment, n (%)^b^	103 (5.3)	53 (3.9)	50 (11.9)	0.001
Depression, n (%)^b^	155 (8.9)	65 (5.2)	90 (25.9)	< 0.001
Female, n (%)	92 (9.6)	37 (5.1)	55 (28.6)	
Male, n (%)	63 (7.9)	28 (5.5)	35 (21.8)	
Depressive symptoms (median, IQR)^a^	2 (0–4)	1 (0–4)	4 (1–10)	< 0.001
Cognitive function (median, IQR)^a^				
CERAD Total	21 (18–24)	21 (18–24)	19 (17–23)	0.001
Delayed Recall	7 (5–8)	7 (5–8)	6 (5–8)	0.004
Animal Fluency	18 (14–21)	18 (15–22)	16 (12–20)	0.021
Digit-Symbol Substitution Test	53 (42–63)	54 (43–64)	46 (32–56)	< 0.001

*CERAD* Consortium to Establish a Registry for Alzheimer's Disease Word List Learning, *BMI* body mass index

^a^Mann-Whitney U-tests were employed to compare the study participants between no walking impairment and walking impairment

^b^Chi-squared tests were employed

The weighted prevalence of visual impairment and depression was 11.9% and 25.9%, respectively in participants with a walking impairment compared to 3.9% and 5.2% respectively in those without a walking impairment (*p* = 0.001). The median cognitive function score across the four cognitive domains was lower in participants with a walking impairment than in those without a walking impairment (*p* < 0.05).

**Fig. 2:**
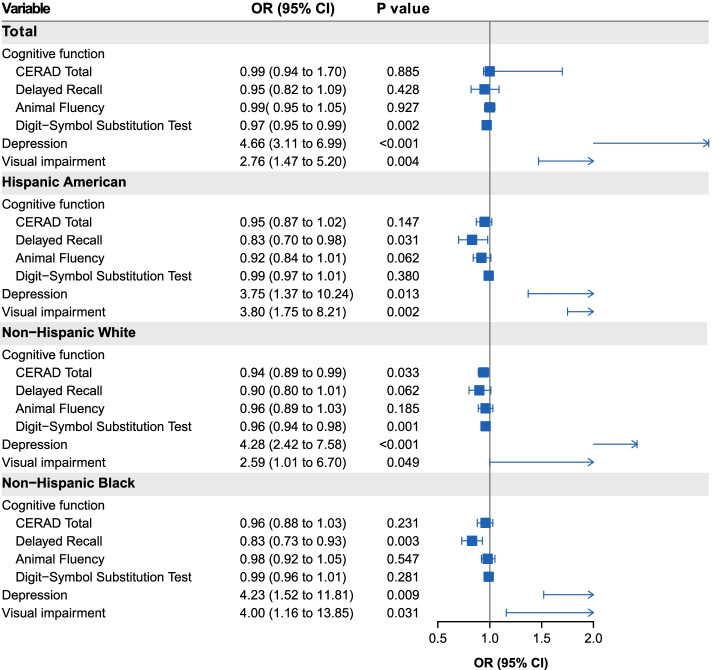
Associations of walking impairment with cognitive function, depression, and visual impairment, NHANES 2013–2014

Table 2 shows the associations of walking impairment with cognitive function, depression, and visual impairment in all the participants stratified by race. Walking impairment was positively associated with the odds of having depression and visual impairment. In the multivariable model, the adjusted odds ratio (aOR) and 95% confidence interval (95% CI) for depression and vision were 4.66 (3.11–6.99) and 2.76 (1.47–5.20), respectively. The cognitive score was inversely associated with walking impairment only for the DSST subdomain (0.97: 0.95–0.99) (Fig. 2).

**Table 2:** Adjusted associations of walking impairment with cognitive function, depression and visual impairment, NHANES 2013–2014

Variables	Model 1	*p* value	Model 2	*p* value	Model 3	*p* value
	OR (95% CI)		OR (95% CI)		OR (95% CI)	
Total						
Cognitive function						
CERAD Total	0.93 (0.90–0.97)	0.002	1.00 (0.95–1.06)	0.883	1.00 (0.94–1.70)	0.885
Delayed Recall	0.87 (0.80–0.95)	0.003	0.95 (0.83–1.10)	0.479	0.95 (0.82–1.09)	0.428
Animal Fluency	0.94 (0.90–0.99)	0.016	0.99 (0.95–1.03)	0.627	1.00 (0.95–1.05)	0.927
Digit-Symbol Substitution Test	0.96 (0.95–0.98)	< 0.001	0.97 (0.95–0.99)	0.002	0.97 (0.95–0.99)	0.002
Depression	5.00 (3.38–7.39)	< 0.001	5.02 (3.36–7.52)	< 0.001	4.66 (3.11–6.99)	< 0.001
Visual impairment	3.24 (1.85–5.65)	< 0.001	3.26 (1.81–5.88)	0.001	2.76 (1.47–5.20)	0.004
By race/ethnicity						
Hispanic American						
Cognitive function						
CERAD Total	0.92 (0.85–0.99)	0.031	0.93 (0.85–1.00)	0.061	0.95 (0.87–1.02)	0.147
Delayed Recall	0.82 (0.72–0.94)	0.007	0.84 (0.73–0.96)	0.015	0.83 (0.70–0.98)	0.031
Animal Fluency	0.91 (0.84–0.98)	0.019	0.91 (0.85–0.97)	0.005	0.92 (0.84–1.01)	0.062
Digit-Symbol Substitution Test	0.98 (0.96–1.01)	0.104	0.98 (0.96–1.01)	0.124	0.99 (0.97–1.01)	0.380
Depression	5.00 (1.95–12.83)	0.002	5.21 (2.01–13.51)	0.002	3.75 (1.37–10.24)	0.013
Visual impairment	6.02 (2.78–13.05)	< 0.001	5.89 (2.49–13.90)	0.001	3.80 (1.75–8.21)	0.002
Non-Hispanic White						
Cognitive function						
CERAD Total	0.93 (0.88–0.99)	0.017	0.94 (0.89–1.00)	0.047	0.94 (0.89–0.99)	0.033
Delayed Recall	0.88 (0.78–0.98)	0.023	0.89 (0.79–1.01)	0.068	0.90 (0.80–1.01)	0.062
Animal Fluency	0.94 (0.89–1.00)	0.055	0.95 (0.89–1.02)	0.118	0.96 (0.89–1.03)	0.185
Digit-Symbol Substitution Test	0.96 (0.94–0.98)	< 0.001	0.96 (0.94–0.98)	0.001	0.96 (0.94–0.98)	0.001
Depression	4.37 (2.54–7.55)	< 0.001	4.45 (2.51–7.87)	< 0.001	4.28 (2.42–7.58)	< 0.001
Visual impairment	2.85 (1.14–7.17)	0.028	2.82 (1.12–7.12)	0.031	2.59 (1.00–6.70)	0.050
Non-Hispanic Black						
Cognitive function						
CERAD Total	0.94 (0.86–1.02)	0.120	0.94 (0.85–1.03)	0.176	0.96 (0.88–1.03)	0.231
Delayed Recall	0.81 (0.70–0.95)	0.010	0.80 (0.68–0.94)	0.009	0.83 (0.73–0.93)	0.003
Animal Fluency	0.97 (0.91–1.03)	0.278	0.97 (0.91–1.04)	0.366	0.98 (0.92–1.05)	0.547
Digit-Symbol Substitution Test	0.97 (0.95–1.00)	0.071	0.98 (0.96–1.01)	0.147	0.99 (0.96–1.01)	0.281
Depression	6.45 (2.31–17.98)	0.002	4.90 (1.74–13.77)	0.005	4.23 (1.52–11.81)	0.009
Visual impairment	3.79 (1.55–9.26)	0.006	4.90 (1.53–15.72)	0.011	4.00 (1.16–13.85)	0.031

Model 1: adjusted for age, sex, race (only for total sample), education, BMI, marital status, sleep disorder

Model 2: Model 1 plus comorbidity (diabetes, hypertension, and stroke)

Model 3: Model 2 plus depression, visual impairment, or cognitive function

In addition, walking impairment was positively associated with depression and visual impairment across the three racial groups. The aORs for depression and visual impairment were 3.75 (1.37–10.24) and 3.80 (1.75–8.21) for Hispanics, 4.28 (2.42–7.58) and 2.59 (1.00–6.70) for Non-Hispanic Whites, and 4.23 (1.52–11.81) and 4.00 (1.16–13.85) for Non-Hispanic Blacks, respectively. The cognitive score was inversely associated with walking impairment only with regard to the Delayed Recall (0.83: 0.70–0.98) for Hispanics, DSST (0.96: 0.94–0.98) and CERAD Total (0.94: 0.89–0.99) for Non-Hispanic Whites, and Delayed Recall (0.83: 0.73–0.93) for Non-Hispanic Blacks.

Table 3 shows the ORs for walking impairment according to the number of impairment indicators (visual impairment, depression, or cognitive impairment). Of the 1,489 participants, 176 participants had two or three impairment indicators, and 90 participants with walking impairment had two or three impairment indicators. Participants with two or three impairment indicators had a higher aOR of walking impairment (aOR = 3.64, 95%CI = 2.46–5.38) than those with 0–1 (reference group) impairment indicator.

**Table 3:** ODD Ratios (ORs) of walking impairment according to number of impairments

Variables	Impairments, NO		*p* value
	0–1	2–3	
Participants, No	1313	176	
Incidence cases, No	230	90	
Model 1, OR (95% CI)	1[Reference]	3.61 (2.45–5.30)	< 0.001
Model 2, OR (95% CI)	1[Reference]	3.64 (2.46–5.38)	< 0.001

Binary logistic regression analysis is presented

Model 1: adjusted for age, sex, race, education, BMI, material statue, sleep disorder

Model 2: Model 1 plus comorbidity (diabetes, hypertension and stroke)

Impairments: Digit Symbol Substitution Test (DSST), visual impairment, depression

DSST impairment: DSST score ≤ 28

## Discussion

Using cross-sectional data from a national sample of older U.S. adults, this study found that walking impairment was associated with visual impairment, depression, and cognitive impairment. Positive associations were found between visual impairment and depression, and an inverse association was found between the DSST score of the cognitive subdomain and walking impairment. Positive associations were also found in all three racial groups, and the associations with cognitive impairment varied by race/ethnicity. In addition, there was also a positive association between the number of impairment indicators and walking impairment.

In our study, the prevalence of visual impairment and depression was higher in older adults with a walking impairment than in those without. The prevalence of walking impairment was 17.5%, the prevalence of vision impairment was 5.3% for all participants and 11.9% for participants with a walking impairment, and the prevalence of depression was 8.9% for all participants and 25.9% for participants with a walking impairment. A previous study estimated that there was a 25% prevalence of visual impairment in individuals aged over 70 [34]. Prevalence of psychological disorders is common among community-dwelling older adults and ranges from 15 to 25% [35]. A cross-sectional study conducted in four community clinics in Moscow among people aged 65 years and older showed that 58.3% of all participants reported visual or hearing impairment, 58.2% reported cognitive impairment, 46% reported mood disorder, and 42% reported difficulty walking [36]. The differences between these studies and ours may be due to the different populations or different definitions of older adults. In addition, depression, visual impairment, and walking impairment were self-reported; therefore, the results may differ from those obtained with clinical data.

In contrast to previous studies [20, 23, 29] we found that visual impairment, cognitive decline, and depression existed individually or coexisted, and were associated with walking impairments in older adults. Furthermore, the associations were stronger between walking impairment and depression than between walking impairment and visual or cognitive impairment in this study. A study explored the relationships between exercise capacity, depression, and cognition in patients with heart failure and found that depression, but not cognition, was independently associated with a walking capacity [28]. The likelihood of this might be supported by the finding that exercise positively affects depression because it contributes to the expansion of brain capacity [37]. Previous studies found that visual impairment was significantly associated with depression, cognitive impairment and walking. Most visual impairments in older adults can be treated, and correcting visual impairment utilizing refractive surgery such as cataract surgery is effective at relieving depressive symptoms [38], highlighting the potential importance of vision screening and managing visual impairments to improve well-being of older adults.

The present study found an inverse association between walking impairment and cognitive score with DSST in older adults. Few studies have examined cognition by measuring multiple fields and their relationship with walking. In a previous study of a nationally representative sample of older U.S. adults using the NHANES (1999–2002), self-reported distance vision dysfunction was associated with poor cognitive function, as reflected in the DSST scores [39]. In a population-based sample of older U.S. adults, distance visual impairment was associated with declining cognitive function (assessed using the Mini-Mental State Examination, MMSE) both cross-sectionally and longitudinally over time [40]. We measured cognitive function across four cognitive domains and not only the DSST or MMSE scores. The cognitive assessments administered in the NHANES 2013–2014 covered four selected domains of cognitive function, and scores from these assessments were not combined to create a composite score with a cut-off that could be used to characterize cognitive impairment, as was done in other studies [23, 41]. It is important to consider multiple cognitive domains since this helps identify people with different types of cognitive impairment, and as such provide tailored care and support. Furthermore, we found that the inverse associations between walking impairment and cognitive subdomains varied by race, with lower scores of Delayed Recall in Hispanics and Non-Hispanic Blacks, and lower scores of DSST and CERAD Total in Non-Hispanic Whites, which is inconsistent with previous studies [26, 27] that suggested that race/ethnicity contributes to individual differences in neural function and related cognitive performance. More research is needed to understand this racial divergence and to aid in identifying interventions to maintain cognitive function and walking across subgroups.

Thus far, studies assessing the association between walking impairment and the combined impairment indicators (vision, depression, and cognitive function) in older adults are sparse.

We found a positive association between the number of impairment indicators and walking impairment. Approximately 28% of the participants with a walking impairment had two or three impairment comorbidities. Similar to a previous study, the prevalence of comorbid depression in older adults with visual impairment was estimated to be approximately 30% [29]. Respective and combined effects of impairments in vision and cognition on gait performance were found in a cross-sectional study, and combinations of impairments in distance vision plus and cognition were associated with an increase in pTUG time (performed Timed Up & Go) [42]. However, abnormal scores on the S-MMSE and the clock drawing test were not sufficient to diagnose satisfactorily memory and executive impairments in this study. Such effect could be explained by the fact that vision, mood, and cognition are key components of walking ability. Our findings have public health implications, since screening for impairment indicators or providing any vision and mental healthcare is beneficial to older adults with walking impairments.

This study has several strengths. First, to our knowledge, this is the largest population-based study to examine the respective and combined associations of visual and mental impairments with walking impairment in older adults. Second, this study examined racial differences. It provides new information on the associations of walking impairment with visual impairment, depression, and cognitive impairment among older adults where data currently does not exist. As for recommendations for clinical practice, these findings could be useful for the development of new preventive and curative interventions dedicated to the improvement of walking impairment in older adults. Understanding the relationships of walking impairment with psychological and visual impairments, all common in older adults, particularly in relation to their racial/ethnic background, is an important research goal as it can inform efforts to reduce health disparities through tailored interventions.

There are some limitations to this study. First, visual impairment and depression were self-reported. Therefore, the results may be different from those obtained using clinical data. Second, this study had a cross-sectional design, so conclusions about the direction of causality could not be made. Given the high prevalence of walking impairment in older adults and the increasing proportion of older adults in the general population, further research is needed to determine whether screening and therapies for visual impairment, depression, and cognitive impairment could help mitigate a decline in walking impairment. Third, few studies have reported the associations of walking impairment with vision, depression, and cognitive function in older adults, leading to a difficulty in thoroughly comparing our results with other findings. Finally, the results of the race/ethnicity subgroup analysis can only be partly extrapolated, but race/ethnicity subgroups still have a certain value in sampling. The research data regarding race/ethnicity provided in this exploratory study is required and important for public health. Further studies are needed to support the racial and ethnic disparities found.

## Conclusion

In the present study, we observed that walking impairment was significantly associated with visual impairment, depression, and cognitive impairment in older adults. An inverse association between walking impairment and cognitive score differed by race. There was also a positive association between walking impairment and the number of impairment indicators. The study suggests that an evaluation of vision, cognitive, and psychological states are needed for older adults with walking impairments while considering their ethnic/racial background.

## Supplementary Information

**Additional file 1: Supplementary Table 1.** Adjusted associations of walking impairment with cognitive function, depression and visual impairment by gender.

## Data Availability

The original NHANES dataset to support this study is available from the National Center for Health Statistics: https://wwwn.cdc.gov/nchs/nhanes/

## Abbreviations

U.S.: United States
NHANES: National Health and Nutrition Examination Survey
aOR: Adjusted odds ratio
CERAD: Consortium to Establish a Registry for Alzheimer's Disease
AF: Animal Fluency
DSST: Digit Symbol Substitution
BMI: Body mass index
NCHS: National Center for Health Statistics
CAPI: Computer-assisted personal interview
IQRs: Interquartile ranges
SDs: Standard deviations
