# Supplementary material for: Associations of walking impairment with visual impairment, depression, and cognitive function in U.S. older adults: NHANES 2013–2014
Source: BMC Geriatr. 2022 Jun 6;22:487. doi: 10.1186/s12877-022-03189-y (PMC9169344; doi:10.1186/s12877-022-03189-y)
Supplement: Supplementary file 1 — Additional file 1: Supplementary Table 1. Adjusted associations of walking impairment with cognitive function, depression and visual impairment by gender. [file 12877_2022_3189_MOESM1_ESM.docx]

Supplementary Table 1. Adjusted associations of walking impairment with cognitive function, depression and visual impairment by gender

| **Variables** | **Model 1** |  | **Model 2** |  | **Model 3** |  |
| --- | --- | --- | --- | --- | --- | --- |
|  | **OR (95% CI)** | ***P* value** | **OR (95% CI)** | ***P value*** | **OR (95% CI)** | ***P* value** |
| **Men** |  |  |  |  |  |  |
| Cognitive function |  |  |  |  |  |  |
| CERAD Total | 0.78 (0.70-1.14) | 0.004 | 0.90 (0.70-1.14) | 0.341 | 0.89 (0.88-1.03) | 0.333 |
| Delayed Recall | 0.95 (1.04-1.12) | 0.197 | 1.03 (0.95-1.11) | 0.453 | 1.03 (0.94-1.12) | 0.507 |
| Animal Fluency | 1.05 (0.97-1.14) | 0.197 | 1.05 (0.97-1.14) | 0.182 | 1.05 (0.96-1.15) | 0.248 |
| DDST | 1.06 (1.04-1.08) | <0.001 | 1.06 (1.04-1.08) | <0.001 | 1.05 (1.04-1.07) | <0.001 |
| Depression | 4.37 (2.26-8.44) | <0.001 | 4.99 (2.77-8.98) | <0.001 | 4.51 (2.47-8.21) | <0.001 |
| Vision impairment | 3.47 (1.25-9.66) | 0.020 | 4.04 (1.35-12.12) | 0.016 | 3.39 (1.21-9.50) | 0.023 |
| **Women** |  |  |  |  |  |  |
| Cognitive function |  |  |  |  |  |  |
| CERAD Total | 1.06 (0.99-1.12) | 0.089 | 1.04 (0.98-1.12) | 0.196 | 1.04 (0.97-1.11) | 0.230 |
| Delayed Recall | 1.10 (0.99-1.23) | 0.072 | 1.08 (0.96-1.21) | 0.207 | 1.07 (0.95-1.20) | 0.257 |
| Animal Fluency | 1.07 (1.00-1.15) | 0.053 | 1.07 (0.99-1.14) | 0.075 | 1.05 (0.98-1.13) | 0.147 |
| DDST | 1.03 (1.01-1.06) | 0.017 | 1.03 (1.00-1.05) | 0.040 | 1.03 (1.00-1.05) | 0.049 |
| Depression | 5.37 (3.41-8.46) | <0.001 | 5.11 (3.09-8.43) | <0.001 | 4.80 (2.86-8.06) | <0.001 |
| Vision impairment | 3.43 (1.72-6.84) | 0.002 | 3.13 (1.48-6.63) | 0.006 | 2.70 (1.12-6.49) | 0.029 |

Model 1: adjusted for age, sex, race, education, BMI, marital status, sleep disorder

Model 2: Model 1 plus comorbidity (diabetes, hypertension, and stroke)

Model 3: Model 2 plus depression, visual impairment, or cognitive function.

**[Abbreviation](https://fanyi.so.com/?src=onebox#abbreviation):** CERAD Total: Consortium to Establish a Registry for Alzheimer's disease Word List Learning; Delayed Recall: CERAD delayed recall; AF: Animal Fluency; DSST: Digit Symbol Substitution.
